# Molecular determinants of signal transduction in tropomyosin receptor kinases

**DOI:** 10.1002/2211-5463.70135

**Published:** 2025-10-06

**Authors:** Giray Enkavi

**Affiliations:** ^1^ Department of Physics University of Helsinki Finland

**Keywords:** allosteric modulation, conformational dynamics, functional selectivity, neurotrophin receptors, transmembrane signaling, tropomyosin receptor kinases

## Abstract

Tropomyosin receptor kinase (Trk) receptors are essential regulators of neuronal development, survival, and plasticity through their interactions with neurotrophins. This review examines the structural and molecular mechanisms connecting ligand binding to the diverse signaling outcomes of Trk receptors. We analyze how neurotrophin binding and allosteric interactions trigger conformational changes that activate distinct signaling pathways. Our discussion explores how allosteric modulation—binding of ligands to sites distinct from the primary receptor site—and ligand bias—where different neurotrophins binding the same receptor preferentially activate certain downstream pathways—may together shape receptor function, focusing on structural and conformational mechanisms. Despite recent advances, important structural details remain unresolved. Further insights into Trk receptor structure and dynamics could significantly enhance therapeutic development by enabling the design of drugs that selectively target‐specific signaling pathways.

## Graphical abstract

Tropomyosin receptor kinases control critical neuronal functions, but how do the same receptors produce diverse cellular responses? This review explores the structural mechanisms behind Trk signaling diversity, focusing on allosteric modulation and ligand bias. Understanding how these mechanisms shape the conformational landscape of flexible receptor domains and thereby modulate signaling outcomes can offer new therapeutic opportunities for neurological disorders.

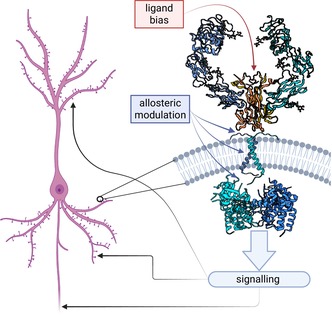

**Abbreviations**
Akt: protein kinase B
ATP: adenosine triphosphate
AXXXG: alanine‐any‐any‐any‐glycine amino acid motif
Bad: Bcl‐2‐associated death promoter
BDNF: brain‐derived neurotrophic factor
Ca^2+^
: calcium ion
CARC: cholesterol recognition/interaction amino acid consensus
CNS: central nervous system
DAG: diacylglycerol
ECD: extracellular domain
EGFR: epidermal growth factor receptor
eJMD: extracellular juxtamembrane domain
EM: electron microscopy
ERK1/2: extracellular signal‐regulated kinase 1/2
ERK5: extracellular signal‐regulated kinase 5
FoxO: forkhead box O
FRET: fluorescence resonance energy transfer
FRS2: fibroblast growth factor receptor substrate 2
Gab1: Grb2‐associated binder 1
GPCR: G‐protein‐coupled receptor
Grb2: growth factor receptor‐bound protein 2
GSK3β: glycogen synthase kinase 3 beta
GTP: guanosine triphosphate
ICD: intracellular domain
Ig‐like domain: immunoglobulin‐like domain
iJMD: intracellular juxtamembrane domain
IP_3_
: inositol 1,4,5‐trisphosphate
IRS‐1: insulin receptor substrate 1
JMD: juxtamembrane domain
KD: kinase domain
LSD: lysergic acid diethylamide
LTP: long‐term potentiation
LXXFAXXF: leucine‐any‐any‐phenylalanine‐alanine‐any‐any‐phenylalanine amino acid motif
MAPK: mitogen‐activated protein kinase
MEK: mitogen‐activated protein kinase kinase
mTOR: mechanistic target of rapamycin
NGF: nerve growth factor
NMR: nuclear magnetic resonance
NT‐3: neurotrophin‐3
NT‐4: neurotrophin‐4
NTRK1: neurotrophic tyrosine kinase receptor type 1 gene
NTRK2: neurotrophic tyrosine kinase receptor type 2 gene
NTRK3: neurotrophic tyrosine kinase receptor type 3 gene
p75NTR: p75 neurotrophin receptor
PAM: positive allosteric modulator
PDB: Protein Data Bank
PDK1: phosphoinositide‐dependent kinase‐1
PI3K: phosphoinositide 3‐kinase
PIP₂: phosphatidylinositol 4,5‐bisphosphate
PIP₃: phosphatidylinositol (3,4,5)‐trisphosphate
PKC: protein kinase C
PLCγ1: phospholipase C gamma 1
Raf: rapidly accelerated fibrosarcoma protein kinase
Ras: rat sarcoma protein
RBA: radioligand binding assay
RTK: receptor tyrosine kinase
SH2: Src homology 2
Shc: Src homology 2 domain‐containing transforming protein
SHP‐2: Src homology region 2 domain‐containing phosphatase‐2
SOS: son of sevenless
SPR: surface plasmon resonance
TIRF: total internal reflection fluorescence
TMD: transmembrane domain
Trk: tropomyosin receptor kinase
TrkA: tropomyosin receptor kinase A
TrkB: tropomyosin receptor kinase B
TrkC: tropomyosin receptor kinase C
UniProt: Universal Protein Resource

Trk (Tropomyosin receptor kinase) receptors play essential roles in the development, maintenance, and function of the nervous system. They are part of the receptor tyrosine kinase (RTK) family, which also includes receptors like the epidermal growth factor receptor (EGFR) and the insulin receptor [1, 2]. Trk receptors are activated by neurotrophins—growth factors such as nerve growth factor (NGF), brain‐derived neurotrophic factor (BDNF), neurotrophin‐3 (NT‐3), and neurotrophin‐4 (NT‐4). There are three primary Trk receptors: TrkA, TrkB, and TrkC [1]. These three receptors evolved from an ancestral proto‐Trk gene through gene duplication events during early vertebrate evolution [3].

While Trk receptors primarily activate conserved signaling pathways common to receptor tyrosine kinases (Ras/MAPK, PI3K/Akt, PLC‐γ), the specificity and diversity of their biological outcomes are largely determined by cellular context, spatiotemporal dynamics of receptor activation, and co‐receptor interactions [1]. This functional diversity is achieved through allosteric modulation, where binding at secondary sites or intramolecular contacts alters receptor function and ligand bias, where different neurotrophins binding to the same receptor selectively activate distinct downstream pathways [4, 5]. This raises a fundamental question: How does a limited set of receptors produce the specific signaling patterns needed for complex nervous system development and function? The answer appears to lie in the structural dynamics and conformational flexibility of these receptors.

This review examines how different binding events—whether through orthosteric neurotrophin binding or allosteric interactions with small molecules—propagate conformational changes through Trk receptors to influence kinase domain arrangements and subsequent signaling patterns. We focus on the structural and conformational aspects of two key mechanisms that expand signaling diversity: allosteric modulation, particularly through juxtamembrane and transmembrane domains, and ligand bias. Understanding these molecular mechanisms provides insight into both normal neurobiological processes and pathological conditions, while offering new approaches for therapeutic intervention in neurological disorders, pain conditions, and cancer.

## Overview of Trk receptor biology and function

### TrkA

TrkA (Tropomyosin Receptor Kinase A) is encoded by the NTRK1 gene [1]. It functions as the high‐affinity receptor for nerve growth factor (NGF) [1, 6]. When NGF binds to TrkA, it activates downstream signaling cascades including Ras/MAPK, PI3K/Akt, and PLC‐γ pathways [2, 6, 7]. These signals promote neuronal survival, differentiation, axonal growth, and synapse formation [6, 8]. TrkA plays crucial roles in pain sensation, with activation leading to nociceptive signaling [9]. Clinically, TrkA is significant in inflammatory pain conditions [10], and NTRK1 gene fusions can drive oncogenesis in various cancers [11, 12]. Mutations in NTRK1 cause congenital insensitivity to pain with anhidrosis, a rare hereditary sensory and autonomic neuropathy [13].

### TrkB

TrkB (Tropomyosin Receptor Kinase B) is encoded by the NTRK2 gene and serves as the receptor for brain‐derived neurotrophic factor (BDNF) and neurotrophin‐4 and can also interact with neurotrophin‐3 (NT‐3) with lower affinity [2, 14, 15]. It is expressed throughout the central nervous system, particularly in the cerebral cortex, hippocampus, and cerebellum [16]. TrkB signaling is fundamental for synaptic plasticity, long‐term potentiation, dendritic growth, and neuronal survival and plays essential roles in learning, memory formation, and mood regulation [14, 16, 17, 18]. TrkB dysfunction has been implicated in various neuropsychiatric disorders, including depression, anxiety, and neurodegenerative diseases [19, 20].

### TrkC

TrkC (Tropomyosin Receptor Kinase C) is encoded by the NTRK3 gene and primarily binds neurotrophin‐3 [1, 2]. TrkC is involved in the development of sensory and motor neurons, contributing to both central and peripheral nervous system functions [21]. TrkC signaling is critical for proprioception (body position sensing), vestibular function, and cochlear development [22] and contributes to enteric nervous system development [23]. Like other NTRK genes, NTRK3 fusions can drive oncogenesis [24].

While highly homologous in sequence, structure, and function, the specific biological processes Trk receptors influence differ. Table 1 summarizes the binding affinities of neurotrophin ligands for each Trk receptor and highlights the biased signaling outcomes. Neurotrophins bind not only to Trk receptors but also, with lower affinity, to the p75 neurotrophin receptor (p75NTR), which belongs to the tumor necrosis factor receptor superfamily [25]. p75NTR binds all mature neurotrophins on its own and forms a complex with sortilin to recognize proneurotrophins—the inactive precursors of neurotrophins that promote cell death signaling [26]. In contrast, as a co‐receptor for Trk receptors, p75 enhances neurotrophin affinity and supports prosurvival and trophic signaling [27, 28, 29, 30]. This dual role allows p75 to fine‐tune neurotrophin responses during development and in the mature nervous system [29].

**Table 1:** Neurotrophin–Trk receptor interactions and their downstream signaling outcomes. Reported binding affinities are not comprehensive and may vary depending on the method and cell line used. The table also lists representative cellular responses elicited by different ligands binding to TrkA, TrkB, and TrkC. SPR, Surface Plasmon Resonance; RBA, Radioligand Binding Assay.

Receptor	Ligand	Affinity	Biased signaling outcomes
TrkA	NGF	*K*_d_ = 1.9 nm (SPR) [31] Low‐affinity site: *K*_d_ = ~1.9 nm; high‐affinity site: *K*_d_ = ~0.01 nm (RBA) [31]	Primarily activates the Ras/MAPK pathway through mainly ERK signaling, resulting in enhanced proliferation and cellular growth [32] Triggers endocytosis, recruits Actin‐modifying proteins, and drives retrograde signaling critical for survival [33, 34]
	NT‐3	*K*_d_ = 130 nm (SPR) [31]	Activates TrkA locally, fails to induce retrograde signaling, so it cannot sustain survival signals [33, 34]
TrkB	BDNF	*K*_d_ = 0.99 nm (RBA) [35] *K*_d_ = 0.79 nm (SPR; recombinant) [36]	Activates Ras–ERK, PI3K, and PLCγ pathways, resulting in neuronal differentiation and survival [32] Favors synaptic plasticity and cognition [37]
	NT‐4	*K*_d_ = 0.26 nm (SPR; recombinant) [36]	Preferentially activates PI3/AKT pathway, blocking apoptosis and boosting cell survival [32] Promotes neuronal survival and maturation [38, 39]
	NT‐3	Lower than BDNF and NT‐4 [40]	Primarily survival signaling, especially early in development [40]
TrkC	NT‐3	*K*_d_ = 0.02 nm [41]	Preferentially activates of the PI3/AKT pathway, preventing apoptosis and increasing cell survival Cell survival, anti‐apoptotic signaling, proprioceptive development

Neurotrophin interactions with Trk receptors are differentially modulated by p75. For TrkA, p75 enhances NGF specificity while reducing responsiveness to NT‐3 [29, 42, 43]. For TrkB, p75 restricts activation primarily to BDNF, excluding NT‐3 and NT‐4/5 [29, 44]. For TrkC, the effect of p75 is less clear [29]. Overall, p75 acts as a differential modulator of Trk receptors, enhancing both affinity and selectivity through receptor‐specific mechanisms [29]. However, the molecular mechanism by which p75 modulates Trk affinity and signaling remains unclear and occurs through different mechanisms: for TrkA, the presence of p75NTR facilitates formation of high‐affinity NGF binding sites [45], while TrkB first binds BDNF independently, then associates with p75 only after receptor phosphorylation [46]. In a 2022 review [29], the authors discuss several models for how p75 modulates Trk activity: (i) heterocomplex formation stabilized by p75, (ii) p75‐mediated ligand concentration within the local microenvironment, and (iii) direct p75‐Trk interaction resulting in allosteric modulation for high‐affinity receptor formation. They also propose a new “inside‐out allosteric modulation” model to describe how p75's intracellular domain promotes high‐affinity Trk binding—a mechanism we briefly revisit later along with other allosteric effects.

TrkA and TrkC function as dependence receptors in peripheral neurons of the sympathetic and sensory systems, meaning they can trigger apoptosis when not bound by their neurotrophin ligands [47, 48]. In contrast, TrkB, predominantly expressed in central nervous system (CNS) neurons, does not act as a dependence receptor, not inducing cell death in the absence of its ligand [48]. This difference helps explain why neurons in the central nervous system do not depend on growth factors for survival to the same extent as neurons in the peripheral nervous system. From an evolutionary perspective, as the nervous system became more specialized with distinct central and peripheral components, the diversification of Trk receptors created a new way to control precisely how many neurons survive in the peripheral nervous system based on the availability of specific growth‐promoting molecules.

These functional differences between Trk receptors also highlight the potential for therapeutic targeting. Beyond their canonical neurotrophin ligands, Trk receptors can be modulated by various small molecules and non‐conventional binding partners for therapeutic purposes [12, 14, 49, 50, 51, 52]. These alternative ligands often interact with nontraditional binding sites, including transmembrane domains [14, 53, 54, 55, 56], and can selectively influence specific signaling pathways. Such interactions can produce unique downstream effects distinct from those triggered by neurotrophins, presenting opportunities for targeted therapeutic interventions in neurological disorders [17, 19], pain conditions [51, 57], and cancer [1, 11, 12].

## Overall architecture and activation mechanism

**Fig. 1:**
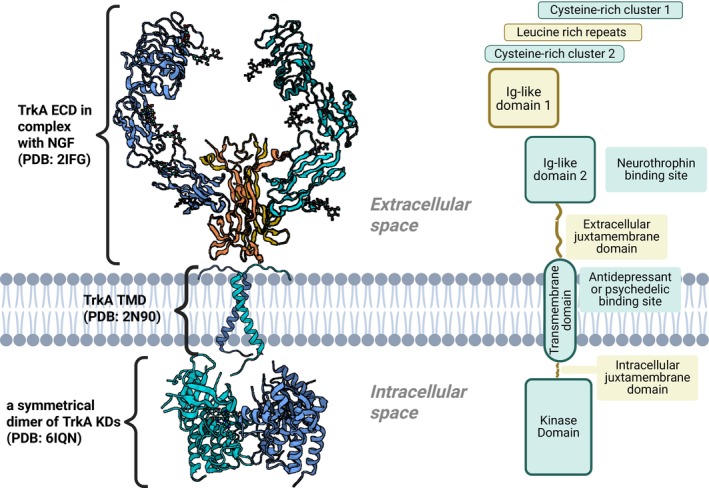
Architecture of Trk Receptors. Left: Trk receptor dimer structure assembled from available crystal structures using TrkA as an example– extracellular domain in complex with NGF (PDB: 2IFG) [61], transmembrane domain (PDB: 2N90) [60], and symmetrical kinase domain dimer (PDB: 6IQN) [62]. Right: Schematic representation of conserved Trk family domains. ECD, extracellular domain; Ig‐like domain, immunoglobulin‐like domain; KD, kinase domain; NGF, nerve growth factor; PDB, Protein Data Bank; TMD, transmembrane domain; TrkA, tropomyosin receptor kinase A.

Trks share the overall structural features associated with RTKs (Fig. 1) [55, 58, 59]. They have an extracellular ligand‐binding domain (ECD), a single‐pass transmembrane helix (transmembrane domain, TMD), and an intracellular tyrosine kinase domain (KD). Trk receptors display sequence and structural homology across their approximately 800 amino acid length. The ECD, comprising the N‐terminal ~400 residues, functions as the neurotrophin binding region and exhibits a defined domain architecture: two cysteine‐rich clusters flanking three leucine‐rich repeats followed by two immunoglobulin‐like domains. This leucine‐rich repeat motif represents a distinguishing characteristic of Trk receptors among receptor tyrosine kinases [36, 59]. A helical transmembrane domain (TMD) of approximately 40 residues connects the ECD to the intracellular region [56, 60]. The intracellular KD contains five functionally critical tyrosine residues: three tyrosine residues positioned within the kinase activation loop regulate catalytic activity, while two tyrosine residues located adjacent to the kinase domain function as phosphorylation‐dependent binding sites for cytoplasmic adaptor proteins and enzymes that mediate neurotrophin signaling cascades [1].

Upon neurotrophin binding, KDs undergo autophosphorylation, initiating downstream signaling cascades. This is driven by structural rearrangements and/or receptor dimerization, which brings the two kinase domains into proximity [63, 64].

Indeed, dimerization alone is likely sufficient for activation, as Clary and Reichardt showed that antibody‐induced cross‐linking of TrkA, in the absence of ligand, elicited NGF‐like cellular responses. Each KD consists of a C‐lobe and an N‐lobe, along with an activation loop. Dimerization lifts autoinhibition, triggers trans‐autophosphorylation whereby kinase domains phosphorylate specific tyrosine residues in their partner's activation loop (Y676, Y680, and Y681 in human TrkA [UniProt: P04629], with Y702, Y706, Y707 in human TrkB [UniProt: Q16620] and Y705, Y709, Y710 in human TrkC [UniProt: Q16288]) [1, 65]. This leads to subsequent phosphorylation of additional tyrosine residues (Y496 and Y791 in human TrkA [UniProt: P04629], with Y516 and Y817 in human TrkB [UniProt: Q16620] and Y516 and Y834 in human TrkC [UniProt: Q16288]), creating docking sites for cytoplasmic adaptors and enzymes [1, 65].

### Preformed dimers in Trks

Like other RTKs, Trk receptors were previously thought to activate through ligand‐induced dimerization. However, compelling evidence demonstrates their existence as preformed, inactive dimers on cell surfaces before neurotrophin binding. Mischel *et al*. [66] using freeze‐fracture EM in Xenopus oocytes, observed transmembrane particles larger than expected for TrkA monomers and found that NGF did not alter their size or shape during signaling. Later, Shen and Maruyama demonstrated that TrkA exists as preformed, yet inactive, homodimers in living cells using chemical cross‐linking, bimolecular fluorescence complementation, and luciferase fragment complementation assays [67]. Their subsequent study extended these findings to TrkB, showing that it also forms preformed dimers in the endoplasmic reticulum before trafficking to the cell surface [68]. The extent of preformed dimerization, however, may depend on the receptor type. Diffusion studies indicate approximately 70% of TrkA molecules appear monomeric, with 20% existing as dimers or oligomers [63]. Moreover, single particle tracking and TIRF microscopy show that ligand binding increases slow dimeric populations over fast‐moving monomeric ones [60, 69]. On the other hand, quantitative FRET measurements [64] reveal TrkB possesses the strongest dimerization propensity among all RTKs studied, with approximately 80% of TrkB molecules forming dimers. TrkA and TrkC demonstrate intermediate dimerization tendencies compared to other receptors under identical conditions. Based on these findings, Paul *et al*. [64] proposed a general transition model for RTK activation wherein receptors form dimers even without ligands, with these unliganded dimers exhibiting variable stability. Upon ligand binding, stabilization of RTK dimers and induction of critical structural rearrangements lead to activation [60, 64].

## Signaling pathways activated by Trk receptors

Like other RTKs, Trk receptor signaling is more complex than a simple on/off mechanism and involves a network of interactions with feedback loops, crosstalk, and context‐dependent modulation [70, 71, 72]. Trk receptor activation initiates three major downstream signaling cascades through phosphorylation‐dependent adapter protein recruitment: Ras/MAPK, PI3K/Akt, and PLCγ1/Ca^2+^ pathways [73, 74]. Each pathway controls distinct cellular processes, from survival and proliferation to synaptic plasticity and differentiation.

### The RAS/MAPK pathway

The RAS/MAPK pathway represents the primary growth‐promoting signal downstream of Trk activation. Phosphorylation of the juxtamembrane tyrosine (Y496 in TrkA, Y516 in TrkB, Y522 in TrkC) creates a docking site for Shc adapter proteins, which undergo subsequent tyrosine phosphorylation by the Trk KD [7, 75]. Phosphorylated Shc recruits the Grb2‐SOS complex, leading to Ras‐GTP loading and sequential activation of Raf, MEK, and ERK1/2 kinases. ERK1/2 phosphorylates diverse substrates, including transcription factors, cytoskeletal proteins, and cell cycle regulators, promoting both immediate responses and long‐term transcriptional changes [76]. In neuronal contexts, sustained ERK activation drives neurite outgrowth and differentiation, while transient activation promotes survival and proliferation [77]. The signaling adapter FRS2 competes with Shc for binding to the same phosphotyrosine site but provides prolonged ERK activation through recruitment of Grb2 and SHP‐2 phosphatase [78] FRS2‐mediated signaling preferentially promotes neuronal differentiation over proliferation.

### The PI3K/Akt pathway

The PI3K/Akt pathway activation occurs through indirect recruitment mechanisms involving multiple adapter proteins. Grb2‐associated binder 1 (Gab1) and insulin receptor substrate 1 (IRS‐1) are recruited to Trk complexes through interactions with Grb2 and undergo tyrosine phosphorylation, creating docking sites for PI3K regulatory subunits [79, 80]. PI3K generates PIP3, which recruits and activates PDK1 and Akt kinases. Activated Akt phosphorylates numerous substrates, including Bad, FoxO transcription factors, GSK3β, and mTOR, promoting cell survival, protein synthesis, and glucose metabolism [81, 82]. In neurons, Akt signaling is essential for NGF‐mediated survival of sympathetic neurons and BDNF‐promoted hippocampal neuron survival. TrkC demonstrates preferential PI3K pathway coupling compared to TrkA and TrkB, contributing to its specialized role in proprioceptive neuron survival during development [83].

### The PLCγ1/Ca^2+^ pathway

The PLCγ1/Ca^2+^ pathway is initiated when the C‐terminal docking site in Trk receptors (Y785 in TrkA, Y817 in TrkB, Y823 in TrkC) recruits PLCγ1 through its tandem SH2 domains [84]. Upon binding, Trk kinases phosphorylate PLCγ1 at Y783, activating its catalytic activity. Activated PLCγ1 hydrolyzes phosphatidylinositol 4,5‐bisphosphate (PIP_2_) to produce inositol 1,4,5‐trisphosphate (IP₃) and diacylglycerol (DAG). IP₃ triggers Ca^2+^ release from endoplasmic reticulum stores, while DAG activates protein kinase C (PKC) isoforms [85]. This pathway controls activity‐dependent processes, including long‐term potentiation (LTP), gene transcription through calcium‐responsive elements, and synaptic plasticity. TrkB Y817F mutants, where PLCγ1 binding is impaired, demonstrate that this pathway regulates synaptic function rather than survival [86].

Downstream signaling networks exhibit extensive crosstalk that integrates signals from multiple phosphorylation sites. PKC activated by the PLCγ pathway can phosphorylate Raf, creating positive feedback between calcium and MAPK signaling [87]. Additionally, ERK can phosphorylate and modulate PLCγ activity, while Akt signaling influences ERK pathway components through multiple mechanisms. The three pathways exhibit distinct kinetics, with MAPK activation occurring within minutes, PI3K/Akt signaling sustained over hours, and calcium responses showing both rapid transients and sustained elevations [88]. This temporal segregation enables complex cellular responses where immediate survival signals are followed by differentiation programs. Recent proteomics studies reveal that pathway outcomes depend on relative phosphorylation stoichiometry across multiple sites, with cells integrating quantitative information from all pathways to determine fate decisions [89].

Recent optogenetic studies have provided direct evidence for how specific phosphorylation patterns control signaling outcomes. Zhao *et al*. [90] used light‐sensitive tyrosine analogues to selectively phosphorylate individual tyrosine residues in TrkA's KD, demonstrating that site‐specific phosphorylation can activate the MAPK/ERK pathway independent of NGF binding. This approach revealed that different phosphorylation patterns of the five critical tyrosine residues produce distinct downstream responses, with certain sites preferentially activating survival pathways while others promote differentiation signals. These findings demonstrate that the phosphorylation pattern, not just overall receptor activation, determines the specific signaling cascade and resulting cellular phenotype.

## Allosteric modulation

Allosteric modulation of Trk receptors represents a promising approach for therapeutic intervention in neurotrophin signaling pathways [91]. Unlike direct agonism or antagonism at the orthosteric binding site, allosteric modulators bind to distinct regions away from the natural ligand binding pocket, allowing for more selective control of receptor function. Two particularly promising targets for allosteric modulation of Trk receptors are the juxtamembrane regions and the transmembrane domains (TMDs). These structural elements play crucial roles in transmitting signals across the membrane and regulating receptor activation states, making them ideal sites for therapeutic targeting with small molecules that can fine‐tune receptor responses without completely blocking or overstimulating the signaling pathway.

A recent review discusses the positive allosteric modulators of Trk receptors in the context of Alzheimer's disease [91]. These modulators target intracellular regions of the receptor to fine‐tune signaling without fully activating or inhibiting the receptor. For example, E2511, a selective TrkA‐PAM, binds to the iJMD of TrkA and exhibits biased signaling by preferentially enhancing Y785 phosphorylation—linked to the PLCγ pathway—over Y490 phosphorylation associated with MAPK/PI3K pathways. However, in another cellular context, the same compound increases phospho‐ERK1/2 and phospho‐ERK5 levels but has no effect on phospho‐PLCγ [92]. ACD856, a pan‐Trk PAM, also binds the intracellular domain of TrkA and increases overall kinase efficiency [93], enhancing TrkB and ERK1/2 phosphorylation [94]. It additionally initiates a feed‐forward mechanism by boosting BDNF levels following TrkB activation. These examples illustrate how targeting non‐catalytic intracellular elements can enable pathway‐specific modulation of receptor activity.

### Juxtamembrane regions (JMD)

In Trk receptors, like other receptor tyrosine kinases (RTKs), the major functional domains—ECDs, TMDs, and KDs—are connected by flexible loop regions. These intrinsically disordered connecting segments, the juxtamembrane domains (JMD), are critical for receptor function and represent viable targets for selective therapeutic intervention. This was first demonstrated for the intracellular juxtamembrane region (iJMD) through structural characterization of TrkA‐selective inhibitors binding to a non‐active site formed by the conserved KD and the non‐conserved JMD [95, 96]. The binding site is highly selective for TrkA due to low sequence conservation between TrkA, TrkB, and TrkC JMDs. These compounds can therefore potentially modulate downstream signaling selectively, including pain‐associated pathways, and may have applications in cancer treatment [95].

Furthermore, the extracellular juxtamembrane (eJMD) region of TrkA was shown to bridge ligand binding to TMD activation by transmitting conformational changes from ligand binding to the TMD [97], switching it between inactive and active states—despite being intrinsically disordered. Similarly, in TrkB, the eJMD serves as a critical regulatory element. It exerts an inhibitory effect on dimerization, which is relieved upon brain‐derived neurotrophic factor (BDNF) binding [98]. Similar functional roles of intrinsically disordered regions have been observed in other RTKs, for example, in computational studies of the KIT receptor [99, 100], where multiple disordered domains including the JMD exhibit conformational plasticity crucial for allosteric regulation and signal transmission between remote functional sites.

Recent clinical advances in Trk receptor modulation highlight iJMDs as promising targets for therapeutic intervention. This binding location allows compounds to influence downstream signaling without interfering with neurotrophin binding at the orthosteric site. Guo *et al*. [101] demonstrated that the iJMD of TrkA forms a unique allosteric pocket for a TrkA allosteric inhibitor. In a recent review, Forsell *et al*. [91] discussed positive allosteric modulators of Trk receptors in the context of Alzheimer's disease [91]. For instance, E2511, a TrkA‐selective positive allosteric modulator (PAM), binds to the iJMD and induces biased signaling by enhancing Y791 phosphorylation—linked to the PLCγ pathway—over Y496, which activates MAPK and PI3K pathways. In other cellular contexts, E2511 increases ERK1/2 and ERK5 phosphorylation but does not affect PLCγ [92]. ACD856, a pan‐Trk PAM, also binds the intracellular domain of TrkA and increases overall kinase efficiency [93], and enhances ERK1/2 phosphorylation [94]. These examples illustrate how targeting non‐catalytic intracellular elements can enable pathway‐specific modulation of receptor activity.

### Transmembrane domain (TMD)

**Fig. 2:**
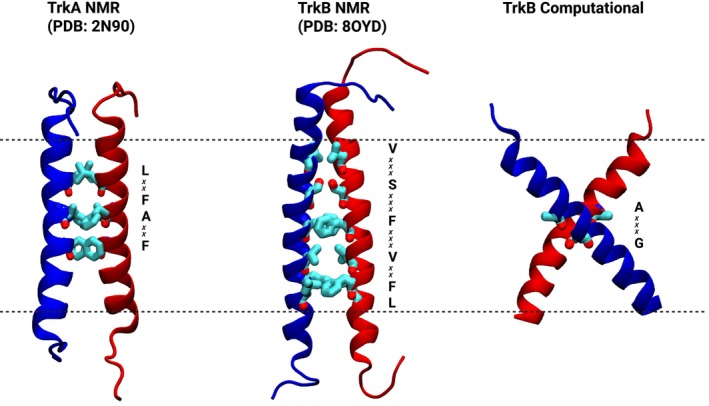
TrkA and TrkB TMD structures. Left: left‐handed TrkA dimer from NMR (PDB: 2N90) [60]. Middle: left‐handed TrkB dimer from NMR (PDB: 80YD) [97]. Right: right‐handed TrkB computational model. Blue and red ribbons depict the two transmembrane helices, with interfacial amino acids shown in cyan licorice representation. Dashed lines indicate approximate membrane boundaries. NMR, nuclear magnetic resonance; PDB, Protein Data Bank; TMD, transmembrane domain; TrkA, tropomyosin receptor kinase A; TrkB, tropomyosin receptor kinase B.

Recent studies have revealed the critical functional role of transmembrane domains (TMDs) in neurotrophin receptors [14, 55], similar to other RTKs [102]. The TMD structure itself consists of α‐helical segments that are structurally identical across all three Trk receptors. However, key structural differences arise in the dimerization patterns of the TMDs: TrkA and TrkB exhibit different dimer conformations, while TrkC TMD dimer structure remains to be determined (Fig. 2).

TrkA was the first Trk receptor whose TMD dimer structure was solved [60]. This structure, determined by NMR spectroscopy, revealed a left‐handed dimer with a ~40° crossing angle and stabilized by the conserved dimerization motif LXXFAXXF [60]. Based on functional studies, it was proposed that the TMD interface of TrkA undergoes a transition from an inactive to an active configuration upon ligand binding, with specific juxtamembrane residues repositioning to enable receptor activation [60]. The LXXFAXXF dimerization interface is highly conserved across species and, while TrkC's TMD dimer structure has not yet been experimentally determined, sequence analysis reveals that TrkC contains the same LXXFAXXF motif found in TrkA, suggesting similar left‐handed dimerization geometry. In contrast to both TrkA and TrkC, the LXXFAXXF dimerization sequence is absent in TrkB. Recent studies suggested two conflicting models for TrkB's TMDs (Fig. 2). One, identified via computational modeling and molecular dynamics simulations, features a left‐handed homodimer adopting a cross‐like conformation that engages the canonical AXXXG dimerization motif [54]. The other, based on NMR studies, reveals a right‐handed dimer with parallel TMD helices stabilized by an extended dimerization interface centered on residue S441 [56]. The NMR structure contradicts the computational model, with mutagenesis experiments confirming that S441A/S441I mutations drastically inhibit BDNF‐induced TrkB activation while AXXXG motif mutations show no biological effects [56]. Based on these functional data, the NMR study proposed that their structure represents the active receptor state, while the computationally predicted AXXXG‐mediated dimer corresponds to an inactive preformed state. However, it is conceivable that the different structural models represent distinct active states that the TrkB dimer can assume, as observed in other RTKs, and that these complementary conformations may be influenced in their stability by factors such as membrane composition.

Regardless of the specific dimerization mechanism, both computational and experimental approaches have revealed important insights into TrkB's TMDs. Bioinformatics and computer simulations suggested the TrkB TMD monomer might interact directly with cholesterol through a CARC domain (particularly via residue Y433) [103]. However, cholesterol modulates the conformation of the dimer and signaling activity via its effects on membrane thickness [54]. Most remarkably, TrkB's unique TMD structure creates a binding site for various antidepressants [54] and psychedelic compounds [53], such as fluoxetine, imipramine, ketamine, LSD, and psilocybin, which act as allosteric potentiators of BDNF signaling, enhancing neuronal plasticity in an activity‐dependent manner. These compounds enhance phosphorylation of TrkB at Y816 (corresponding to Y785 in TrkA) and increase the interaction between TrkB and PLCγ1 [53, 54]. The computational model, mutagenesis, and NMR studies agree on the key binding residues, with fluoxetine showing specific interactions at residues Y434 and V437 [53, 54, 56]. This binding site is absent in TrkA and TrkC, making TrkB a distinctive pharmaceutical target for mood disorders through its TMD [53, 54]. We refer the reader to our recent review for a more detailed discussion of TrkB TMD in ref [14].

TMDs play a critical role in Trk–p75NTR interactions, and receptor‐specific variations in Trk TMDs may determine how each Trk interacts with p75NTR, contributing to the dependence receptor behavior observed for TrkA and TrkC, but not TrkB. Structural evidence indicates that TMDs mediate complex formation between p75 and TrkA. Several interfacial residues are conserved in TrkB and TrkC, suggesting that p75NTR can form TMD‐mediated complexes with these receptors [104]. Moreover, Conroy *et al*. [29] proposed an “inside‐out allosteric mechanism,” where the p75 TMD stabilizes or modulates the TrkA TMD conformation to promote low‐affinity activation. Meanwhile, the p75 intracellular domain (ICD) interacts with the TrkA kinase domain (KD), triggering an allosteric conformational change in the KDs. This change then propagates through the TMDs to the extracellular domain (ECD), priming the receptor for high‐affinity binding [29].

The critical role of JMDs and TMDs in Trk receptor function highlights their significance as regulatory elements in signal transduction. The JMDs are intrinsically flexible and lack a defined structure. Likewise, TMD dimers exhibit flexibility by adopting multiple packing arrangements and orientations in response to the membrane environment and ligand binding. These regions form specific binding pockets that accommodate small molecules with high specificity, suggesting that conformational dynamics are essential for allosteric modulation. Their ability to adopt multiple conformations allows fine‐tuning and modulation of signaling by stabilizing certain conformational states, although this flexibility also complicates structural characterization.

## Diversity of signaling outcomes and ligand bias

The same receptor can lead to diverse biological outcomes. While allosteric modulation is one manifestation of this variability, ligand bias is another. Also termed functional selectivity or biased agonism, ligand bias refers to the capacity of different ligands to preferentially activate distinct signaling pathways despite binding to the same receptor [5, 72, 105]. Ligand bias has been extensively characterized in G‐protein‐coupled receptors (GPCRs) [106, 107, 108, 109] but for receptor tyrosine kinases (RTKs), it is less well characterized and presents distinct challenges [5]. Ligand bias can only be assessed by comparing a ligand's effects across multiple signaling pathways, as this reveals how it preferentially activates certain pathways [110]. This contrasts with the pharmacological concepts of potency and efficacy, which are concerned with a ligand's strength and maximal effect on one specific signaling outcome [110, 111]. Various methods, such as bias plots, offer a data‐driven approach to quantify ligand bias based on dose–response curve parameters [112].

Ligand bias has influenced drug development, as seen in the growing number of biased ligands approved for clinical use, especially for treating conditions like pain (opioid receptors), heart failure (β‐adrenergic receptors), and metabolic disorders (insulin and glucagon receptors) [106, 113, 114, 115]. In Trk receptors, small‐molecule agonists can exploit biased signaling to selectively activate trophic pathways, highlighting their therapeutic potential for neurodegenerative diseases [116]. Ibáñez *et al*. [117] showed that point mutations in neurotrophins can yield ligands with similar Trk phosphorylation but distinct biological effects, suggesting that ligand structure can bias signaling despite comparable receptor activation. Chen *et al*. [118] provided early empirical evidence for biased agonism in Trk receptors by synthesizing selective TrkC peptidomimetics that selectively activated distinct TrkC‐mediated pathways: some enhanced survival without neurite outgrowth, while others induced neuritogenesis without trophic effects.

Resende‐Lara *et al*. [119] proposed that biased signaling in TrkA receptors results from ligand‐ or mutation‐induced changes in receptor conformational dynamics. Using normal mode analysis—a computational method that identifies collective protein motions—they showed that the R221W mutation in nerve growth factor (NGF), linked to congenital insensitivity to pain, suppresses receptor motions associated with nociceptive signaling while preserving those tied to differentiation. This computational study highlights structural modulation of TrkA as a mechanistic basis for allosteric and biased signaling [119]. How these altered dynamics translate into specific downstream pathway activation remains to be elucidated.

In a recent review, Samad *et al*. (2024) [72] discuss ligand bias in RTKs from a systems‐level perspective, emphasizing the roles of ligand identity, ligand concentration, receptor crosstalk, and spatiotemporal regulation. Different ligands trigger specific conformational changes, activating divergent pathways despite identical receptor engagement. Ligand concentration operates through threshold‐dependent switches, enabling transitions between cellular states. Receptor crosstalk—whether between different RTKs or with other plasma membrane molecules—modulates signaling and determines the final signaling outcome. Spatiotemporal regulation occurs within subcellular compartments such as the plasma membrane and endosomes, each providing distinct biochemical environments that affect signaling outcomes. Similarly, receptor trafficking—whether degradation or recycling—determines signaling duration and pathway selectivity. Together, these factors turn simple signals into complex cellular decisions, contrasting with molecular‐level ligand‐receptor interactions.

At the single‐molecule level, ligand identity is key to linking receptor conformations to diverse signaling outcomes, with various mechanisms potentially at play. One such mechanism is kinetic proofreading [120, 121], where the stability of active receptor complexes determines the recruitment of downstream signaling proteins. This process involves a time delay between receptor–ligand binding and the formation of stable complexes, allowing for the dissociation of unstable complexes or their dephosphorylation by phosphatases to ensure productive signaling [120]. The lifetime of these complexes directly influences signaling outcomes. The EGFR system illustrates this principle: [120, 122] certain ligands induce weaker dimers, promoting sustained signaling that leads to differentiation, while others trigger more stable dimers, resulting in rapid internalization and transient signaling that drives proliferation [120, 122]. A similar principle applies to Trk receptors, which exhibit ligand bias: Stable Trk‐A dimers promote neuron survival and growth, while weaker dimers support axon growth but not neuron survival [4]. Using FRET and fluorescence intensity fluctuation spectroscopy, Ahmed *et al*. [4] showed that NGF stabilizes TrkA dimers more effectively than NT‐3. Like kinetic proofreading in EGFR [120, 122], more stable NGF‐TrkA dimers may engage feedback mechanisms leading to receptor downregulation. Since TrkA signaling regulates both neuronal survival and axon growth, the varying stability of receptor–ligand complexes likely contributes to biased signaling outcomes. p75NTR further influences these responses, as discussed earlier, by enhancing NGF specificity and suppressing NT‐3–dependent TrkA activation [29, 42]. Mischel *et al*. demonstrated that p75NTR co‐expression completely blocks NT‐3‐induced TrkA signaling through ligand‐independent conformational control requiring the ECD [43], while in the absence of p75NTR, NT‐3 can substitute for NGF in supporting neuronal survival [123].

**Fig. 3:**
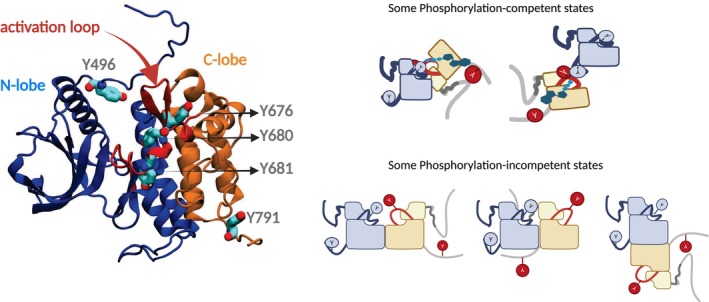
Trans autophosphorylation requires precise positioning of activation loop tyrosines relative to the ATP‐binding active site. Left: TrkA KD (alphafold model) showing the N‐lobe (blue) and C‐lobe (orange) with the flexible activation loop (red) containing critical tyrosine residues (Y676, Y680, Y681) that must be phosphorylated for full kinase activation, along with signaling tyrosines Y496 and Y791. Right: Schematic representation of phosphorylation‐competent states (top) where KD arrangements allow activation loop tyrosines to access the active sites of opposing protomers for reciprocal phosphorylation, versus phosphorylation‐incompetent states (bottom) where domain orientations prevent productive substrate presentation. ATP, adenosine triphosphate; C‐lobe, C‐terminal lobe; KD, kinase domain; N‐lobe, N‐terminal lobe.

For TrkB, ligand binding results in both differential dimer stability and the adoption of multiple conformations, which together contribute to diverse signaling outcomes and ligand bias. FRET measurements show that different ligands, including NT‐3, NT‐4, and BDNF, induce distinct relative positioning and dynamics of the TrkB domains, suggesting that different ligands stabilize distinct kinase dimer configurations (Fig. 3) [124]. Thermodynamic data further reveal that the stability of TrkB dimers varies depending on the ligand, with BDNF‐bound dimers exhibiting the highest stability. These findings suggest that both the structural flexibility of the dimer and its thermodynamic stability are key mechanisms underlying the ligand‐specific signaling bias in TrkB receptors [124].

Despite the complexity involved, RTK signaling outcomes are fundamentally determined by kinase domain (KD) configurations, which direct downstream pathway activation. Distinct phosphorylation patterns produced by KD activity can engage with downstream partners in specific ways, resulting in diverse signaling outcomes and underlying ligand bias. As a structural framework, Karl and Hristova [125] proposed the concept of RTK dimer state, which comprises an ensemble of microstates, each characterized by a set of distinct KD dimer configurations for the same ECD structure. For phosphorylation of a specific target Tyr, the active site of the partner KD must establish proper contact with it. The phosphoryl transfer reaction requires an optimal distance of less than 4 Å between γ‐phosphate of ATP and the hydroxyl group of Tyr [126] (Fig. 3). When KDs arrange in this precise configuration, they form a phosphorylation‐competent microstate. Any alternative configuration results in phosphorylation‐incompetent microstates, which cannot catalyze phosphorylation. A KD dimer microstate ensemble is composed of phosphorylation‐competent and phosphorylation‐incompetent microstates, each of which might have a unique phosphorylation pattern.

Structural bioinformatics has identified 15 phosphorylation‐competent kinase domain dimer complexes in the Protein Data Bank [127]. Trans autophosphorylation involves diverse KD arrangements observed in crystal structures, including face‐to‐face dimers with symmetric or asymmetric activation loop exchange, back‐to‐back dimers, and various activation loop exchange conformations. Symmetrical arrangements are not required – asymmetric configurations can form, and multiple energetically favorable dimer interfaces can exist [128].

In this model [125], the prevalence of a particular microstate in the ensemble depends on the restraints imposed on it by the other RTK domains. Ligands or allosteric modulators can change the conformation of ECDs or TMDs and shift the distribution of KD microstates. It can be envisioned that within the ensemble, the probability of forming a particular phosphorylation‐competent microstate for a given tyrosine influences its likelihood of phosphorylation, ultimately leading to a distribution of phosphorylation states.

This dynamic ensemble of KD microstates provides a mechanistic explanation for how different ligands or regulatory conditions can generate distinct signaling outcomes from the same receptor. Ligand binding modifies the receptor's conformational landscape, selectively enhancing the probability of certain phosphorylation‐competent microstates while suppressing others. This leads to a unique phosphorylation pattern, which facilitates specific docking of downstream signaling proteins, ultimately guiding pathway‐specific cellular responses. Thus, the microstate ensemble model [125] links receptor‐level structural dynamics to selective pathway activation and distinct biological outcomes. Understanding the structural configurations of the relevant KD microstates and the overall receptor conformations that lead to them would be highly beneficial for therapeutic and drug design purposes. However, this information is currently lacking.

## Concluding remarks

Trk receptor signaling is a complex molecular process that extends beyond simple on/off switches. The receptors' conformational flexibility allows them to produce diverse signaling outcomes. Allosteric modulation, particularly in the juxtamembrane and transmembrane regions, plays a key role in shaping receptor behavior. Ligand bias, where different ligands stabilize distinct receptor conformations, results in differential phosphorylation patterns and selective activation of downstream pathways. Despite significant progress, key structural details remain unresolved, particularly regarding how orthosteric or allosteric ligand binding is coupled with changes in kinase domain interactions. Computational approaches offer a promising direction for understanding Trk receptor dynamics. Integrating experimental findings with computational modeling can reveal a more complete conformational landscape that governs signaling diversity. This combined approach can enable the structure‐based design of precisely targeted therapeutics for Trk‐mediated disorders, offering more effective treatments for neurological disorders, pain, and cancers linked to Trk receptor dysfunction. These insights could lead to drugs that selectively modulate specific signaling pathways, minimizing off‐target effects.

## Conflict of interest

G.E. has ownership interests and employment relationships with Kasvu Therapeutics, Inc., which had no influence on the contents of this manuscript.

## Author contributions

GE conceived and designed the review, conducted the literature analysis, and wrote the manuscript.
